# Dubowitz Syndrome: A Review and Implications for Cognitive, Behavioral, and Psychological Features

**DOI:** 10.4021/jocmr581w

**Published:** 2011-07-26

**Authors:** Rebekah S. Huber, Daniel Houlihan, Kevin Filter

**Affiliations:** a University of Utah, SLC, USA; b Minnesota State University, Mankato, USA

## Abstract

Dubowitz syndrome is a rare autosomal recessive disorder characterized by micorcephaly, short stature, abnormal faces, and mild to severe mental retardation. Growth retardation occurs both intrauterine and postnatal. Behavioral characteristics include hyperactivity, short attention span, and aggressiveness. Behavior problems include difficulty feeding, sleep disturbance, and bedwetting. Individuals with the disorder have displayed shyness, fear of crowds, and dislike of loud noises. A high-pitched or hoarse voice is common. Deficits have been found in speech and language skills, reasoning and memory skills, self-help skills, and psychomotor functioning. Ocular, dental, cutaneous, skeletal, cardiovascular, gastrointestinal, neurological, immunological, and hematological medical difficulties have been noted. Approximately 148 cases have been described in the literature. The cause of the disorder remains unknown, however, research suggests genetic origin. Past research emphasizes physical characteristics and medical complications. There is a lack of cognitive, behavioral, and psychological information available regarding the disorder. This article presents a review of the literature and provides assessment and treatment implications for the cognitive, behavioral, and psychological aspects of Dubowitz syndrome.

**Keywords:**

Dubowitz; Syndrome; Autosomal; Recessive

## Introduction

Dubowitz syndrome is a rare autosomal recessive disorder that was first identified in children over 40 years ago. Less than 200 cases have been documented in the literature. It is marked by growth retardation, microcephaly, short stature, characteristic facial features, skin eruptions, and mild to severe mental retardation [1]. Children with the disorder also display similar behavioral characteristics. Hyperactivity and short attention span [2], impulsivity and aggressiveness [3, 4], shyness and a dislike of crowds [5], refusal of food and bedwetting [3] have all been reported. Certain medical problems are associated with the condition [6-10]. Dubowitz syndrome commonly leads to decreased motor and language functioning [5], developmental delay [3], and an increased risk for a shorter life span [4, 6, 13].

The disorder was first discovered in a female child in 1965 by Victor Dubowitz. He described the disorder and proposed it was a phenotype of Boone’s syndrome [1]. The syndrome was later identified and named by Grosse [2] and Opitz [11]. The cause of Dubowitz syndrome remains unknown, however, literature points to genetics and chromosomal instability [3, 12, 13]. Sporadic cases suggest growth hormone deficiency [14] or defects in the cholesterol biosynthetic pathway [15] as the source of the disorder. Due to its rarity, most reports discussed in the literature are case studies that emphasize specific physical anomalies and new medical findings.

Emphasis has been given to physical and medical phenomenology of the disorder, but there is a lack of thorough examination of cognitive, behavioral, and psychological aspects. To date, only one publication has focused specifically on the psychological status of individuals with Dubowitz syndrome [3]. The study reported on intellectual functioning, language and fine motor developmental delays in ten cases. Parent report addressed adaptive functioning and behavioral deficits [3]. However, this research was conducted more than two decades ago and failed to objectively evaluate individuals’ behavioral characteristics and adaptability. There is a need in the literature to measure these features to provide a complete illustration of the disorder and how it affects children. The literature also fails to discuss management of these attributes. This paper thoroughly reviews 148 cases of Dubowitz syndrome and provides assessment and treatment implications for cognitive, behavioral, and psychological functioning.

## Description of Disorder

### Phenomenology

#### Facial characteristics

Individuals with Dubowitz syndrome have distinct facial characteristics which distinguish it from other disorders with similar symptomology. Distinctive abnormalities with the shape of the face, eyes, ears, nose, and mouth are common. The face is narrow or triangular shaped [5, 18] with a sloping forehead [5, 16, 17]. The supraorbital ridge is shallow and even with the face [5, 17, 18]. Micrognathia, a receding chin that results in an underbite, was present in 85 of the cases [5, 16-19]. The hair and the eyebrows are thin and sparse [5, 10, 16, 18, 19]. Facial asymmetry or abnormal neck has been reported, but occurs in less than ten percent of cases [5]. The ears vary in size, but are usually more prominently positioned. Ears are low set [5, 19] and can be posteriorly angulated [5]. In 41 cases, dysplastic (poorly-formed) ears exist [10]. Otis media (inflammation of the middle ear) is often severe and can require the insertion of tubes [24]. A broad or flat nasal bridge is common [5, 10, 16], and some cases document a prominent round tip of the nose [5].

Several malformations are associated with the eyes. Drooping of the upper eyelid was observed in the majority of cases [5, 10, 16, 18]. Epicanthal folds are excessive and cover the inner corner of the eye [5, 18]. Twenty five percent of the cases report abnormal palperbral fissures [5, 17, 19] (narrow longitudinal opening above the eyelids). Fifteen percent noted telecanthus [5, 17, 18] (increased distance between the inside corner of the eye to the nose) or hypertelorism [5, 16]. Investigation of the size and functioning of the eyes has also revealed irregularities. Unnatural smallness of the eyes has been mentioned [5, 18]. Microphthalmia (large eyes in comparison to the face) were noted in one case [18]. Megalocornea (enlarged rear segment of the eye) is rare [20]. Strabismis and estropia exist but have been less mentioned [5]. Visual problems such as farsightedness (hyperopia), cataracts, and primary pigmentary degeneration of the retina (tapetoretinal degeneration) have also been found [5, 20]. Iris coloboma, (some structure of the eye is absent) iris hypoplasia, (underdevelopment of the iris), nystagmus, anisocoria, paresis, and astigmatism were noted in less than 5 cases [5, 20]. Strabismus is an anomaly in which the eyes are not aligned with each other under normal conditions [20]. Children who experience strabismus can also experience diplopia (double vision) and problems with depth perception due to the misalignment of the eyes. Often, one of the images is suppressed by the brain in order to compensate [20].

A large mouth is rare, but was observed in some cases [5]. Studies have shown multiple abnormalities with the palate [25, 27]. The palate has been found to be unusually narrow and high arched [5, 18]. Other oral abnormalities include cleft palate, submucous cleft palate, and cleft uvula [5, 10]. Velopharngeal insufficiency results in failure of the soft palate to reach the rear pharyngeal wall which can cause high pitched or hoarse speech [5]. Dental manifestations include crowded, missing, irregular, or rotated teeth. In children with Dubowitz syndrome, delayed eruption of teeth has been more commonly documented [5, 18].

#### Growth retardation

Growth retardation occurs both pre and post birth. In 14 cases, it was found only after birth [5]. One of the most distinct features is the small size of the body and the head. Microcephaly, short stature, and low weight is common [5, 10, 16-19]. In 141 cases, the average circumference of the head at birth was 31.0 cm and the weight ranged from 900 to 3,600 g [5]. Short stature is not as common as low weight. In 56 percent of cases, intrauterine growth retardation (birth weight less than 2,500 g at full term) was noted [5]. Physical development is often months to years behind the actual age of the child [3]. Growth charts of 5 cases presented by Orrison and colleagues illustrate growth retardation in all cases [21]. At birth, only one child was 3 standard deviations below normal birth weight. When measured months later, 4 children were below the 3rd standard deviation and one was below the second standard deviation of weight for their age [21].

Retarded bone maturation can result in decreased tone of the skeletal muscle, characterized by weakness and floppiness [10] (hypotonia). Retarded bone maturation was found in over 25% of cases [5, 10]. In a few individuals, the joints were hyper extensible [5, 10]. Other anomalies include scoliosis [22], missing or underdeveloped ribs [10, 16], and fused vertebra [5, 10]. One patient presented skeletal abnormalities similar to his brother who was diagnosed with Klippel Feil syndrome [16]. The hands and feet are not apart from physical deformities. Problems with the feet include flat footedness, club foot, dorsiflexion, plantar flexion, abducted and inverted foot, or various combinations in which the foot is twisted out of shape or position [5, 10]. More common anomalies include fifth finger clinodactyly, which is permanent bending of the “baby” finger, and syndactyly, which the 2nd or 3rd fingers or toes have fusion or webbing together with skin [5, 21]. Two cases found children with abducted thumbs [5, 23].

Dubowitz syndrome has been associated with genital and rectal abnormalities [5, 24, 25]. Cryptorchidism, the condition when one or both testes fails to descend, was found in 27 cases [5, 10]. Hypospadias, abnormal development of the urethra, was found in 10 cases [5]. In males, the urethra opens on the bottom surface of the penis or in the perineum. In females, the urethra opens into the vagina. Genital anomalies in females were found in 23 cases, which included hypospadias or hypoplastic genitalia. This includes an underdeveloped clitoris, labia, or vagina [5]. Chronic diarrhea and constipation have been found in 16% of cases [5]. Illynia and Lurie first noted rectal malformations in patients with Dubowitz syndrome in 1990. Anal stenosis and constipation were discovered in children from 3 different families [25]. Less common anal and rectal difficulties consist of rectal prolapse, anal stenosis, congenital constipation, inguinal and hatial hernia [5, 10, 25].

#### Cognition

Dubowitz syndrome has shown a deficiency in cognition [1]. Mild to moderate mental retardation is common [3, 5]. Severe mental retardation is rare, but has been found in 7% of the cases [5, 25]. Some cases reported that children with cognitive deficits attended special education classes or special schools [3, 5, 6, 22]. Normal intelligence also exists in children with the syndrome [3, 5, 10, 18]. Children with Dubowitz syndrome have also shown a short attention span [3, 7, 18, 24, 25] and preference for concrete thinking [5]. In the evaluation of ten cases, Parrish and Wilroy indentified deficits in reasoning and memory, and severe delays with expressive and receptive vocabulary [3]. Difficulties specific to individual cases included problems with spatial perception, numerical abilities, spelling, abstract reasoning, visual motor coordination, and fine motor development [3]. In the study, many parents also reported that their children were months to years behind other children with regard to self-help skills [3]. A few children required assistance with dressing, eating, and toileting [3].

#### Behavioral characteristics

There are many behavioral characteristics associated with Dubowitz syndrome that have been reported throughout the literature. These are known to affect speech, temperament, and overall behavior. In the majority of patients, there are problems related to language development and speech [3, 5, 10, 15, 20, 24, 26]. A high pitched voice, which is common to the disorder was found in 39 cases [5-7, 19, 26, 27]. High pitched or hoarse voice [22] can be characterized as a voice disorder. Voice disorders are often accompanied by problems with articulation or speech delay which form a complex communication disorder. Development of speech was delayed in 20% of cases [3, 5, 10, 15, 20, 26]. It is important to correctly identify any communication difficulties and get treatment started as soon as possible to prevent other learning or developmental problems [19, 27].

In 53 cases, children exhibited behavior problems [5, 19, 21, 24]. Hyperactivity was found in the 34% of the total cases [5, 7, 19, 24, 25]. Tsukahara and colleagues mentioned that hyperactivity was “very difficult to manage and in some cases interferes with sleep” [5]. Hyperactivity can exacerbate impulsiveness, problem behaviors, and learning deficits. Frequent outbursts are not uncommon in these children [5]. Less reported behavior problems include irritability, agitation, moodiness, carelessness, poor compliance, and frequent tempertantrums [3, 5, 10, 19].

Another behavioral factor that appears to be affected by Dubowitz syndrome is temperament [3, 5, 7, 20, 28]. Although it is somewhat uncommon, children have shown a tendency to withdrawal from others or experience shyness [5, 20, 28]. Other individuals dislike large crowds or have a fear of public places [5]. Individuals with a range of mental abilities prefer to avoid crowds and public places [5, 20]. It has not been distinguished if individuals simply have an aversion to these places or if they are uncomfortable with their physical appearance and consequently avoid public. Children with hyperactivity and impulsivity may also experience anxious [4, 5] or aggressive temperament [5, 7, 10, 25]. Irritable, whiny, and attention seeking has been found [3, 5, 10]. Understanding the temperament of the individual can also help direct treatment and set up an educational program that is appropriate for the child [19, 27].

Difficulty feeding has been found in 31 cases of children with Dubowitz syndrome [4, 5, 9, 12, 21]. These difficulties are sometimes discovered immediately after birth and include food refusal, vomiting, and regurgitation [4, 5, 9, 21]. Some cases with difficulty feeding have found to be associated with medical concerns of the gastrointestinal system or the esophagus [5, 9]. On the other hand, lack of eating has been associated with failure to thrive or anorexic-like tendencies [12, 21, 29].

Failure to thrive is associated with a child’s inability to gain weight, grow or feed normally [29]. Several causes have been hypothesized for the disorder of failure to thrive. One relates low birth weight with the mother’s nourishment during intrauterine development [29]. Another theory proposes that children do not receive effective reinforcement from parents when the feeding behavior occurs [29]. Study of the feeding behaviors of a child diagnosed exclusively with failure to thrive were assessed by Casey and colleagues [29]. Via descriptive analysis, researchers identified schedules of reinforcement by the child’s parents. When the child refused food and the parents removed the food, it resulted in negative reinforcement. The study implemented a behavioral intervention to address feeding behaviors with the child and schedules of reinforcement with the parents. When refusal of food became extinct in the study, normal feeding behaviors that resulted in weight gain and growth persisted [29].

Less common behavior characteristics include a preference for vibrations and musical beat [5]. Children sit close to speakers to enjoy vibrations from playing music [5]. Other fascinations include watching ceiling fans or rolling credits on television, and engaging in repetitious phrases and behaviors [5]. Bouncing, dancing, twirling of the hair, and giggling have all been found to be repetitious, excessive, and often inappropriate [5]. When parents reported on their children’s behavior, a few children displayed sensitivity to criticism, were “picky eaters”, or wet the bed [3].

#### Medical difficulties

Case studies of patients with Dubowitz syndrome have shown various medical phenomena. Medical problems with the skin, esophagus, gastrointestinal system, cardiovascular system, immune system, and neurological problems have all been established [4-9, 23, 26, 27]. Although they don’t appear regularly, they still have shown to exist in cases and are associated with the disorder.

Complications with the integumentary system are common in individuals with this disorder. Fifty-nine patients reported a cutaneous abnormality [5, 7, 26, 27]. Since the disorder was first discovered, eczema has been considered to be a characteristic feature, however, it has been vaguely described in the literature [1, 2, 11]. Eczema is a type of dermatitis that causes acute or chronic noncontiguous inflammation of the skin. It is characterized chiefly by redness, itching, and the outbreak of lesions that may discharge serous matter and become encrusted and scaly [26, 27]. A small amount of cases examined various skin problems in depth [7, 26, 27]. One case study found scaly skin dermatitis in a young boy and made a diagnosis of ichthyosiform eruption [26]. He responded well to a treatment with daily topical application of 10% urea-containing ointment. Fifty percent of individuals reported to have eczematous lesions did not respond to the typical treatment of topical corticosteroids with success [26]. Kato and colleges suggested an incorrect diagnosis of eczema in many of these cases. They implied that some lesions may a result of ichthyosiform eruption [26]. Although rare, keliodal lesions have also been reported in a few cases [5, 7].

Problems with the esophagus and gastrointestinal system are often related to feeding problems. Irregularities found in the literature include gastroesophageal reflux [5, 21] (return of the stomach contents back up into the esophagus), and achalasia [9] (narrowing of the esophagus). Vomiting and regurgitation result with both. Vomiting occurred with 24 of the cases in which 5 of the cases involved gastroesophageal reflux [5]. There is still much information needed to understand the cause of feeding problems and the association to the esophagus and gastrointestinal system.

Medical concerns with the hematology, immunology, and malignancies include aplastic anemia, actute lymphatic leukemia, malignant lymphoma, germinal blastic sarcoma, neuroblastoma, pancytopenia, embryonal rhabdomyosarcoma, hypogammaglobulinmia, hypoplastic anemia, and bone marrow hypoplasia [4-6, 12, 13, 25]. These have occurred very rarely in cases with Dubowitz syndrome. However, with the majority of the anomalies, there is great risk for a fatal outcome [4, 5]. Aplastic anemia results when bone marrow causes sufficient red and white blood cell production [4, 6]. Acute lymphatic leukemia is a rapidly progressing cancer of the blood that affects white blood cells [5]. Lymphoma is malignant tumor of the lymph glands [5]. Neuroblastoma is a malignant tumor derived from primitive ganglion cells that most commonly appear on the adrenal glands [5]. Hypogammaglobulinmia is a condition where the immunoglobulin level is depressed below the normal range. In many of these cases, chromosomal instability and breakage was described [5, 12, 13]. Frequent infections, allergies, and asthma are associated with Dubowitz syndrome. Infections appear in the ears, sinuses, gastrointestinal system, respiratory system, and urinary tract [5, 7, 13, 24].

Other reported medical complications consist of cardiovascular abnormalities, neurological problems, and migraine headaches [5, 8, 10, 17, 23, 28]. Congenital heart defects have been found in 13 cases [5, 23]. The defects included ventricular septal defect, heat murmurs, atrial septal defect, mitral valve prolapse, and coarctation of the aorta [5, 23]. Many of these abnormalities required surgical attention. Neurological difficulties were found in 17% of the cases. Seizures with abnormal and normal EEG findings, migraine headaches, and paralysis of the limbs, anus and bladder have been reported [5, 8, 17, 28]. Abnormalities in the brain have also been discovered [5, 10, 17]. These included decreased density in the frontal region of the brain and abnormal ventricles [5, 10, 17].

### Diagnosis

Diagnosis for Dubowitz syndrome is based primarily on inspection of facial appearance, growth records, and previous medical history [19]. It is difficult to diagnosis, as some of the characteristic facial features are not always prominent or similar in each case [19]. A medical doctor can make a diagnosis of the disorder, however, there are a few doctors that are considered to be experts at diagnosing Dubowitz syndrome [1, 5, 11]. Diagnosis by these experts is not required, however, these doctors have familiarity with the features and have often made more than one diagnosis. Consulting a physician that has had previous experience with the disorder is recommended if symptoms are variable and do not fit the common features of the disorder. At this time, the karotype is normal, so diagnosis is done primarily through observation methods [5, 19]. Patients with Dubowitz syndrome are often evaluated by several different specialists for associated medical problems and abnormalities with eyes, ears, teeth, skin, and skeletal system [19].

#### Age

Diagnosis can occur at any age [11, 19, 22]. Diagnosis is more common during early childhood due to the associated features and medical problems [5, 19]. It has also been diagnosed in adolescence and adulthood [11, 22]. Some infants have been diagnosed with the disorder due to low birth size and weight, microcephaly, and recurrent need of medical assistance following birth due to medical difficulties [4, 5, 19]. It is common that undiagnosed patients present with other medical problems and upon examination, Dubowitz syndrome is diagnosed.

#### Differential diagnosis

Dubowitz syndrome can easily be confused with other disorders. It has frequently been mistaken for Bloom syndrome, Fanconi anemia, and fetal alcohol syndrome [1, 2, 5, 8, 11, 12, 19]. Dubowitz syndrome shares similar symptoms of mental retardation, skin lesions, and growth retardation. Fanconi anemia and Dubowitz syndrome share problems with chromosomal instability and the hematological and immune systems [5, 12, 19]. However, Bloom syndrome and Fanconi anemia do not share the facial characteristics of Dubowitz syndrome and therefore can be easily distinguished [1, 2, 5, 8, 11, 12, 19]. On the other hand, fetal alcohol syndrome has similar facial abnormalities, hyperactivity, growth retardation, varying degrees of mental retardation, and microcephaly [5, 8, 19]. Fetal alcohol syndrome requires intrauterine exposure to alcohol which separates it from Dubowitz syndrome [5, 8, 19]. Dubowitz syndrome and fetal alcohol can also be differentiated by evaluating clinical manifestations, as they are different in each disorder [5, 8, 19].

### Prevalence

#### Gender

Dubowitz syndrome is present equally in males and females. Of the 148 cases reported, 66 were female and 73 were male [5, 10, 16-19]. The literature failed to report the gender of nine cases [5].

#### Race and location of origin

The disorder has been found in diverse groups worldwide. Sixty-nine percent of the cases have been reported in the United States, Russia, and Germany [5]. Cases have also been found in Japan [16], Spain [19], Turkey [17], Hong Kong [18], France, Italy, Mexico, Canada, England, Poland, Venezuela, Israel, Hungary, Croatia, Netherlands, Brazil, and Poland [5]. The disorder does not appear to be specific to any particular race or region.

#### Family

Dubowitz syndrome has shown to exist within other members of the family. Nine cases of family occurrence are reported in the literature. The disorder has occurred in siblings and twins [1, 2, 5, 11, 24, 25] Dubowitzs original findings describe the disorder in three sisters [1]. Tsukahara and Opitz discovered Dubowitz syndrome in only one of two twins [5]. One case reported of the diagnosis within an aunt [10] and another case described a mother with similar features of the disorder [5].

### Etiology

The cause of Dubowitz syndrome remains unknown. Due to familial occurrence, it is believed to have genetic origins. Direct consanguinity was found in three cases involving parents [1, 5, 13] and one case involving first cousins [27]. Hormone deficiency has been found sporadically throughout the literature [5, 14, 23]. Three cases showed success with growth hormone treatment [5, 14, 23]. Other cases have reported hyperthirodism, iron deficiency, and cholesterol deficiency [5, 15]. Chromosomal instability and breakage has been examined in detail in a few cases [5, 12, 13]. However, Tsukahara and Opitz assert that metabolic and DNA repair defects must be ruled out as the cause [5]. Instead, they suggest that mosaic pleiotropy, which is intrcellular action of the muntant genotype during various periods of pre and post natal development. Their reasoning is based on the broad variability of phenotype that there must be action of many modifying genetic or epigenetic factors [5]. This however, fails to account for the fact that the phenotype is similar among siblings with Dubowitz syndrome [5]. Recent research declares that Dubowitz syndrome is a microdeletion and a microduplication syndrome rather than an autosomal disorder [23].

## Behavioral Assessment/Conceptualization

### Cognitive functioning

Due to the severe physical and medical effects the disorder has on an individual, it is necessary to assess all physical aspects and medical concerns during examination. Past research has shown the benefits of examining different characteristics such as the skin, immune system, brain structure, lymphatic functioning and hormones, skeletal, ocular, dental, and ears. It is equally important to assess the cognitive functioning of individuals. Assessment and diagnosis of cognitive deficits can guide treatment and individualized education plans [19, 27]. Much of the literatures discusse the cognitive and behavioral aspects of each case, however, there is not a great deal of information regarding assessment in the majority of individuals described with cognitive problems.

Few studies have used cognitive and developmental assessments to tap into the functioning of children with Dubowitz syndrome [3, 8, 21, 22]. The Thermann-Merrill was used to asses IQ in one case [22]. Five cases assessed personal, social, fine motor, adaptive, language, and gross motor development with the Denver Developmental Screening [21]. In Parrish and Wilroy’s psychological analysis of ten children, the measures used included the Cattell Infant Intelligence Test, Stanford-Binet Intelligence Scale, Wechsler Intelligence Scale for Children-Revised, Vineland Social Maturity Scale, Peabody Picture Vocabulary Test-Form A, Bender Gestalt Test, Goodenough Draw-A-Man Test, and Wide Range Achievement Test [3]. Sunku and colleagues reported results of one of childs performance on the Wechsler Intelligence Scale [8].

Information is missing from the literature about how cognitive functioning was assessed in many individuals diagnosed with mental retardation. Lack of proper cognitive assessment may be inferred in some of the cases that present mental retardation in Dubowitz syndrome. In the majority of the existing cases with a diagnosis of severely mentally retarded, the literature described developmental and speech delays. However, no assessment was mentioned. This suggests that the child was not tested, but rather assumed to have severe mental retardation based on the inability to talk, walk, or perform specific tasks.

When assessing cognitive abilities, it is essential to use a valid measure that generates an IQ score and level of performance. Suggested assessments that can achieve this task include the Wechsler Intelligence Scales [30-32], Stanford-Binet Intelligence Scale: 4th Edition [33], Bayley Scales of Infant Development [34], and the Test of Nonverbal Intelligence [35]. Individual functioning and academic achievement can be measured on the Wechsler Intelligence Scale for Children 4th Edition (WISC-IV) [30] and the Wechsler Individual Achievement Test 2nd Edition (WIAT-II) [31]. For youth ages 6 to16, the WISC-IV is used [30]. The Wechsler Preschool and Primary Scale of Intelligence (WPPSI-III) [32] is used for children ages 2 7 years, 3 moths. The Stanford-Binet Intelligence Scale: 4th Edition [33] has also been used to properly assess intelligence and provide an IQ score for individuals ages 2 up. For some children, it may not be practical to administer the Wechsler or Stanford-Binet [31, 33]. There is no single assessment that is appropriate for all individuals. The Bayley Scales of Infant Development [34] and the Test of Nonverbal Intelligence [35] can be utilized instead.

A measure of development and performance in various areas would also be essential. This could be accomplished with the Denver Developmental Screening: Revised [36]. Additionally, many individuals diagnosed with mental retardation were in their early years of childhood [5]. It would be important to have an initial measurement of cognitive ability and continue to reassess the IQ, development, and performance of each child. This longitudinal measurement could show change over time and help identify needs for treatment and special educational programs.

### Maladaptive behavior

Behavior of children with Dubowitz syndrome also needs to be assessed. Children have displayed hyperactivity, aggressiveness, difficulty concentrating, behavior problems, sleep disturbance, and feeding problems. The literature fails to discuss an assessment strategy for these individuals. Suggestions for assessment include behavioral observation, functional assessment, behavioral checklists, the Differential Ability Scales [37], and the Vineland Adaptive Behavior Scales [38].

Many of the associated behavioral characteristics such as behavior problems, hyperactivity, aggressiveness, sleeping and feeding difficulties can all be examined through the use of behavioral observation or functional assessment. Functional assessment would provide more information about the antecedents and consequences of the behavior and can guide treatment. Behavioral observation can assess psychomotor retardation. Behavioral checklists such as the Vineland Adaptive Behavior Scales could be given to a parent to complete. It is not suggested that behavioral checklists are administered alone. They need to be supplemented with an objective measure. Functional assessment and observation is one way that this can be accomplished and can be applied to a variety of settings and behaviors. A multifaceted approach to behavioral assessment is recommended, and can be accomplished by incorporating different methods of measurement.

Dubowitz syndrome and attention deficit-hyperactivity disorder (ADHD) have common symptoms of hyperactivity, aggressiveness, irritability, and the inability to concentrate. Due to the overlap of the symptomology, it is important to consider ADHD as a possible diagnosis and to use the proper tools for assessment. The Differential Ability Scales [37] have shown validity in assessment for ADHD. Accurate diagnosis of ADHD would be essential to developing a treatment and educational plan for the individual.

### Speech and language

It is necessary to assess speech functioning to check for social skills deficits in children with Dubowitz syndrome. One case showed that a patient was very outgoing, friendly, attentive, and talkative [18]. Other cases have shown patients to be withdrawn and shy [5, 28]. The range of cognitive abilities, temperament and social skills illustrate the need for further assessment. Speech and language assessment can be measured by the Peabody Picture Vocabulary Test-3rd edition (PPVT-III) [39], Assessment of Child Language Comprehension (ACLC) [40], and Clinical Evaluation of Language Fundamentals-Revised (CELF-R) [41].

## Treatment

### Medical

Various treatments have been used with different medication conditions associated with Dubowitz syndrome. The majority of treatments consist of surgery or medication. Surgery has been done on the face, hands, and feet to correct abnormal features. The skeletal, gastrointestinal, ocular, dental, and cardiovascular systems have also been treated with surgery.

Aphakic extended-wear contact lenses were used to treat a boy with nuclear cataracts and strabismus [20]. In other cases, surgical removal of cataracts and cosmetic strabismus surgery can help restore comfortable, normal, clear vision that also provides an aesthetically pleasing result. Despite the benefits of cosmetic strabismus surgery, common side effects include persistent diplopia.

Other interventions consist of medications, blood transfusions, growth hormones treatments, and transplants. Treatment for aplastic anemia has shown to be most effective with a bone marrow transplant. However, a donor is not always available, so although it has been found to be extremely effective, it is not the most common form of treatment. A blood transfusion might also be beneficial, especially if there are no other treatment options. One case of a young girl was fatal due to aplastic anemia. Her parents denied treatment due to religious convictions and she died three months later as a result of the medical condition [4].

### Cognitive and language treatment

Children with cognitive difficulties can attend special education classes or specialized schools to assist with their learning disabilities. An individual education plan can be developed for each child which emphasizes areas of deficits and needs. By following this educational plan, parents and teachers can help advance a child’s abilities while accentuating strengths. Speech therapy can be utilized to develop language deficits. It can also help children learn how to annunciate and articulate words. Due to the abnormities experienced orally with the palate, instruction of tongue placement and voice projection would be beneficial. It will also be necessary to treat social skill deficits and expand self-help skills for individuals with Dubowitz syndrome. Social skills and self-help skills can be taught through roll play, social play, or reinforcement schedules.

### Behavioral interventions and management

There are many behavioral treatments that can be applied to the associated features of the disorder. After thorough assessment, treatment can be implemented for behavior problems, hyperactivity, feeding difficulties, sleep disturbance, and dislike/fear of public places. Differential reinforcement is can be used as an effective treatment with many of the associated behaviors. It is defined by contingent and appropriate reinforcement based of the desired response and ignoring or not acknowledging the target behavior.

Although medication has proven to be successful with ADHD, behavioral treatments have also shown to be efficacious. Behavioral treatment recommended for hyperactivity, short attention span and behavior problems includes differential reinforcement. Feeding problems associated with Failure to Thrive can also employ differential reinforcement after a thorough functional assessment [29]. Differential reinforcement can increase parental reinforcement and feeding abilities such as bite-acceptance.

Sleep disturbance is often due to physical anomalies and medical conditions, however sleeping difficulties have also been attributed to hyperactivity and behavior problems. It is necessary to carry out a functional assessment in order to determine the underlying cause of sleep disruption. If sleep disturbance is not due to physical or medical manifestations, then a behavioral treatment can be implemented. A behavioral treatment can decrease sleep disturbance and help a child sleep thru the night. Use of differential reinforcement or a token system can encourage more sleep and decrease restfulness. Keeping a sleep log and recording sleep latency, duration, waking episodes and behaviors that accompany them.

## Empirical Status/Outcome Research

There is a lack of information on the long-term outcome of the disorder, associated medical problems, and adult level of functioning. Few cases have been studied longitudinally, and the outcomes of the disorder vary for each individual [5, 28]. Individual outcomes appear to be related to the primary physical, medical, cognitive, and psychological manifestations of the disorder, how they progressed over time, treatment, and the individuals adaptability.

A longitudinal study of the first child diagnosed with Dubowitz syndrome describes childhood, adolescence, and adulthood [28]. The female in the study had mild mental retardation and completed high school in special education classes. She had undergone multiple surgeries, including plastic surgery to her face. She had experienced abnormal menstruation at age 13. At age 22, she endured two seizures for which she was prescribed phenobarbital for two years with no later recurrence. As a child, she had severe eczema that caused sleep disturbance. By adulthood, her eczema had subsided and she was able to sleep through the night. She appeared young for her age and had high tolerance to pain. At the time of evaluation, she was living independently and working full-time in a sheltered workshop [28].

## Implications for Future Research

Future study of children with Dubowitz syndrome should include cognitive, behavioral, and psychological evaluation. Focus should be given to assessment of these features, which can provide specific information to direct treatment, as well build on the current knowledge-base of the disorder. Behavioral interventions that address associated characteristics should be prominent in future research. More research is needed concerning the long-term functioning and adaptability of these individuals. Children with the disorder appear and function differently from peers. It would be essential to evaluate the affects of the disorder on an individuals self-esteem, social, and relational aspects. Much research is needed in regard to the cognitive, behavioral, and psychological status of children with Dubowitz syndrome.

## Summary/Conclusion

 Dubowitz Syndrome is a rare childhood disorder. It has shown to affect individuals physically, medically, cognitively, behaviorally, and psychologically. The serious medical and physical manifestations of the disorder highlight a need for further evaluation of cognitive, behavioral, and psychological aspects of children with Dubowitz syndrome. Review of the literature revealed a lack of thorough evaluation regarding these features. Previous reports provide a record of associated characteristics, but fail to mention assessment and treatment options for behavioral and psychological findings. This paper suggests various assessment and treatment implications concerning cognitive, behavioral, and psychological symptomology. Much research is needed to advance the current knowledge of the disorder and to improve the quality of care for individuals with Dubowitz syndrome.
